# Impact of seasonal blood pressure changes on visit-to-visit blood pressure variability and related cardiovascular outcomes

**DOI:** 10.1097/HJH.0000000000003759

**Published:** 2024-05-01

**Authors:** Giuseppe Mancia, Helmut Schumacher, Michael Böhm, Guido Grassi, Koon K. Teo, Felix Mahfoud, Gianfranco Parati, Josep Redon, Salim Yusuf

**Affiliations:** a University Milano-Bicocca, Milan, Italy; b Statistical Consultant, Ingelheim am Rhein; c Universitätsklinikum des Saarlandes, Homburg, Germany; d Population Health Research Institute, McMaster University and Hamilton Health Sciences, Hamilton, ON, Canada; e Department of Medicine, INCLIVA Research Institute, University of Valencia, Valencia, Spain

**Keywords:** blood pressure variability, cardiovascular risk, seasonal blood pressure, visit-to-visit blood pressure

## Abstract

**Background:**

Visit-to-visit blood pressure (BP) variability associates with an increased risk of cardiovascular events. We investigated the role of seasonal BP modifications on the magnitude of BP variability and its impact on cardiovascular risk.

**Methods:**

In 25 390 patients included in the ONTARGET and TRANSCEND trials, the on-treatment systolic (S) BP values obtained by five visits during the first two years of the trials were grouped according to the month in which they were obtained. SBP differences between winter and summer months were calculated for BP variability quintiles (Qs), as quantified by the coefficient of variation (CV) of on-treatment mean SBP from the five visits. The relationship of BP variability with the risk of cardiovascular events and mortality was assessed by the Cox regression model.

**Results:**

SBP was approximately 4 mmHg lower in summer than in winter regardless of confounders. Winter/summer SBP differences contributed significantly to each SBP-CV quintile. Increase of SBP-CV from Q1 to Q5 was associated with a progressive increase in the adjusted hazard ratio (HR) of the primary endpoint of the trials, i.e. morbid and fatal cardiovascular events. This association was even stronger after removal of the effect of seasonality from the calculation of SBP-CV. A similar trend was observed for secondary endpoints

**Conclusions:**

Winter/summer SBP differences significantly contribute to visit-to-visit BP variability. However, this contribution does not participate in the adverse prognostic significance of visit-to-visit BP variations, which seems to be more evident after removal of the BP effects of seasonality from visit-to-visit BP variations.

## Graphical abstract

**Figure:**
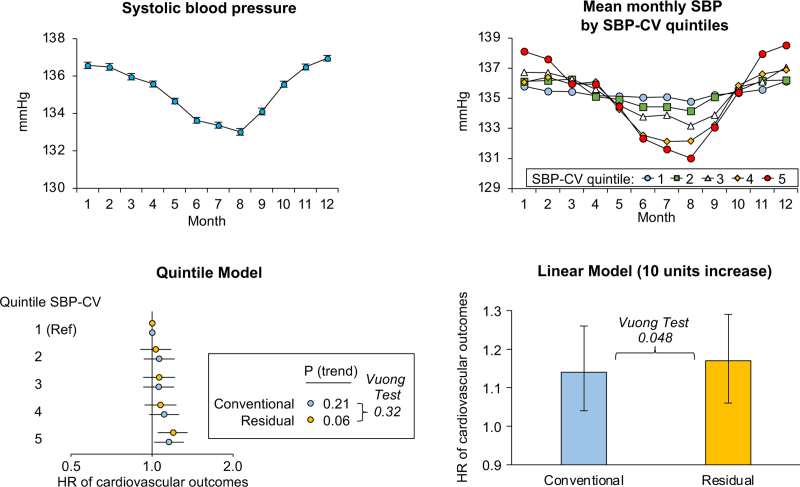

## INTRODUCTION

Evidence has been obtained that the protective effect of antihypertensive treatment depends not only on the average reduction of blood pressure (BP) values during the treatment period but also on the consistency of the BP lowering effect over time. This was shown by the posthoc analysis of several large scale antihypertensive treatment trials, in which the reduction of cardiovascular outcomes was found to be independently associated with the number of visits during which patients achieved BP control [1–3]. It has also been documented by many other studies which showed that, in patients under antihypertensive drugs, BP variability between visits performed at several month intervals was associated with cardiovascular morbid and fatal events independently of the mean BP value during the treatment years [4–15].

The studies that have focused on visit-to-visit BP variability have also provided information on the factors associated with the genesis of this phenomenon. BP variability has been found to be greater when adherence to the prescribed antihypertensive treatment is low [16,17]. In some studies, beta-blockers and calcium channel blockers have been reported to be accompanied by greater and smaller visit-to-visit BP variations, respectively [18–24]. Greater BP variability values have been found in patients with organ damage, including an increase of arterial stiffness [25–27]. Factors such as endothelial dysfunction, smooth muscle reactivity, physical activity, sodium intake, blood viscosity and sleep deprivation have also been postulated [28–31]. In contrast, little attention has been devoted to the possibility that, because visit-to-visit BP variability is quantified via BP measurements performed at several month intervals, its size is influenced by BP seasonality, i.e. the lower BP values that have been reported to occur in summer compared to winter months [32–37], in some studies with differences in cardiovascular morbid events as well [37–41]. Aim of the present study has been to determine whether seasonal BP differences contribute to visit-to-visit BP variability and this contribution plays a role in the adverse prognostic value associated with between visits BP variations. Data were retrieved from the large database made available by the Ongoing Telmisartan Alone and in Combination with Ramipril Global Endpoint Trial (ONTARGET) and the Telmisartan Randomized Assessment Study in ACE Intolerant subjects with Cardiovascular Disease (TRANSCEND) in patients under antihypertensive treatment for 5 years or more [42,43]. Because these trials recruited a large number of patients in countries from different continents, our study also allowed us to pursue a secondary goal, i.e. to analyze on a large scale the BP effects of seasonality in different geographic areas as well as in treated patients with different demographic and clinical characteristics.

## METHODS

### Main trials

The design, methods, and treatment algorithm of the ONTARGET and TRANSCEND trials have been reported in detail previously [42,43]. Briefly, ONTARGET and TRANSCEND were multicenter trials including a total of 31 546 patients with known atherosclerotic disease or diabetes with organ damage. Patients’ recruitment involved 40 countries from various areas of the world (Table S1, Supplemental Digital Content). Individuals with a systolic (S) BP >160 mmHg or a diastolic (D) BP >100 mmHg were excluded. After a single blind run-in period, ONTARGET patients were randomized to take telmisartan once daily, ramipril once daily or both. The daily doses of the two drugs at the end of the titration phase were 80 and 10 mg, respectively. TRANSCEND recruited exclusively patients intolerant to angiotensin converting enzyme (ACE) inhibitors and randomized them to telmisartan (80 mg once daily) or placebo. In both trials patients were allowed to use additional antihypertensive drugs other than ACE-inhibitors or angiotensin receptor blockers if needed. In either trial randomized treatment was continued in a double-blind fashion for a median follow-up of 56 months, during which patients’ visits were planned after 6 weeks and 6 months from randomization and at 6 month intervals thereafter. In both trials the primary outcome was a composite of mortality for cardiovascular causes, nonfatal myocardial infarction, nonfatal stroke and hospitalization for heart failure. Secondary outcomes were the components of the primary outcome, renal outcomes and all-cause mortality. The main objectives of ONTARGET were to determine whether the cardiovascular protection offered by telmisartan and the combination of telmisartan and ramipril were respectively not inferior or superior to that offered by ramipril alone. The main objective of TRANSCEND was to determine whether the cardiovascular protection offered by telmisartan was superior to that offered by a treatment which did not include blockers of the renin-angiotensin system.

### BP measurements

BP was measured in the physicians’ office, using a validated semiautomatic device (HEM 757; Omron, Kyoto, Japan). At each visit a single measurement of BP and heart rate (HR) was obtained after the patient had rested in a sitting position for approximately 3 min. Measurements were made about 24 h after the administration of the prescribed drugs.

### Visit-to-visit BP variability

Details on the calculation of visit-to-visit BP variability have been reported previously [12,13]. Briefly, to measure the extent to which, within any single patient, SBP varied from one visit to another, in each patient the mean and standard deviation (SD) obtained from the 5 visits performed within the initial 2 years of treatment was calculated. SBP values obtained during the titration phase (beginning from the randomization visit) were excluded to avoid inclusion of visit-to-visit SBP variations intentionally determined by the physician to achieve BP control. To be included in the calculations, visits had to be made at least 30 days before an event, to avoid possible BP distortions due to the event proximity. Only patients with exactly five valid visits (resulting in 25 390 patients), were analyzed because visit-to-visit BP variability calculated from five visits has been found to correlate closely with the risk of cardiovascular and renal outcomes [12,13] and using the same number of visits in each patient avoids the instability problems in the calculation of BP variability generated by a variable number of measurements [24]. The SBP-SD was divided by mean SBP and multiplied by 100 to obtain the SBP coefficient of variation (SBP-CV), which was taken as the measure of the intra-individual tendency of SBP to differ between on-treatment visits. As reported in previous studies on ONTARGET and TRANSCEND patients [12,13], SD showed, as expected, a positive correlation with mean BP whereas no correlation was found between SBP-CV and mean SBP. Thus, SBP-CV represents an independent measure of intra-individual visit-to-visit SBP variability at variance from SBP-SD and, in this population, another suggested measure of variability such as the variability independent on the mean or VIM [4]. The study population was subdivided into quintiles of SBP-CV. The average number of visits was similar for the different quintiles both in winter (December to February) and in summer (June to August), i.e. 1.24, 1.22, 1.23, 1.25, 1.27 for winter in quintiles 1 to 5, respectively; and 1.25, 1.24, 1,25, 1.26, 1.30 for summer in quintiles 1 to 5, respectively. In other words, there was no imbalance in the number of winter and summer BP measurements or visits between quintiles. Visit-to-visit DBP variability was not analyzed because most data on between-visit BP variations refer to their systolic component [4–15].

### Data analysis

Data from the three ONTARGET and the two TRANSCEND treatment groups were pooled. In each patient SBP, diastolic BP and HR values were analyzed according to the month in which the measurement had been made. Monthly means were calculated for the group as a whole and for various subgroups (northern and southern Europe, northern and southern hemispheres, males and females, different age groups, diabetic and nondiabetic patients, different baseline SBP ranges, and different on-treatment SBP ranges). In both the group as a whole and in the various subgroups BP data were corrected for the inverse seasonality between northern and southern hemisphere, i.e. in the southern hemisphere the 1st six months of the year were interchanged with the months in the 2nd half of the year of the northern hemisphere (January became July and so on), and adjusted for the baseline covariates listed in Table 1, using the mixed model for repeated measurements (MMRM). A subgroup analysis was also done for SBP-CV quintiles.

**TABLE 1:** Baseline demographic and clinical characteristics of patients. Data are shown for patients with five visits (at least 30 days before a cardiovascular event or death) within the first 2 years, separately for on-treatment SBP-CV quintiles (conventional)

		On-treatment SBP-CV quintiles				
	All	1	2	3	4	5
Number of patients	25390	5078	5084	5072	5078	5078
SBP-CV, %	8.8 ± 4.0	3.9 ± 1.0	6.4 ± 0.6	8.3 ± 0.6	10.5 ± 0.8	15.0 ± 2.8
Northern hemisphere, %	81.3	83.7	82.1	81.4	80.6	78.5
Age, years	66.2 ± 7.1	65.6 ± 7.0	66.5 ± 7.0	66.1 ± 7.1	66.5 ± 7.1	67.2 ± 7.1
Males, %	70.8	72.6	72.1	72.4	69.2	67.6
Whites, %	71.7	75.3	73.8	71.5	69.9	68.0
BMI, kg/m^2^	28.1 ± 4.7	28.3 ± 4.8	28.2 ± 4.7	28.2 ± 4.6	28.0 ± 4.6	27.9 ± 4.7
Baseline SBP, mm Hg	141.5 ± 17.1	141.6 ± 15.9	141.2 ± 16.8	141.4 ± 17.3	141.4 ± 17.3	141.9 ± 18.4
Baseline DBP, mm Hg	82.1 ± 10.3	82.8 ± 9.7	82.3 ± 10.2	82.2 ± 10.1	81.9 ± 10.4	81.5 ± 10.9
Baseline HR, bpm	67.7 ± 12.1	68.7 ± 11.7	67.9 ± 12.1	67.7 ± 12.1	67.2 ± 12.1	67.2 ± 12.3
Serum creatinine, mg/dl	1.05 ± 0.26	1.04 ± 0.25	1.04 ± 0.24	1.05 ± 0.26	1.06 ± 0.27	1.07 ± 0.29
eGFR, ml/min per 1.73 m^2^	74.0 ± 19.6	74.6 ± 19.6	74.9 ± 19.2	74.3 ± 19.4	73.4 ± 19.4	72.6 ± 20.2
Obesity, %	32.6	33.5	32.6	32.6	32.5	31.6
Current smoking, %	11.8	11.3	11.3	12.0	12.2	12.1
Alcohol consumption, %	39.7	41.5	40.2	40.0	40.1	36.9
Physical activity, %						
Mainly sedentary	21.9	22.4	21.7	20.7	21.2	23.3
<once/week	11.1	11.4	12.1	10.2	10.5	11.3
2–6 times/week	31.0	31.9	29.9	31.9	31.8	29.6
Everyday	36.0	34.3	36.3	37.2	36.5	35.8
Hypertension, %	69.7	69.6	68.6	68.9	69.1	72.3
Diabetes mellitus, %	35.9	37.0	34.8	35.5	35.8	36.4
Previous MI, %	48.5	49.0	48.8	47.8	49.2	47.7
Previous stroke/TIA, %	20.3	19.5	19.2	20.2	20.4	22.1
Use of beta blockers, %	58.0	56.2	57.4	56.7	58.5	61.4
Use of diuretics, %	27.3	25.6	26.6	25.8	27.8	30.9
Use of CCBs, %	25.5	26.5	26.0	25.1	25.4	24.4
Participation in ONTARGET, %	81.2	78.9	79.6	81.8	81.6	83.9
Study treatment, %						
Placebo	9.3	11.2	10.5	9.3	8.3	7.3
Ramipril	27.4	26.9	28.3	27.6	27.6	26.5
Telmisartan	36.7	38.5	37.5	36.9	36.2	34.5
Telmisartan + ramipril	26.5	23.4	23.7	26.1	27.8	31.7
Adherence to study treatment, %						
<50%	3.3	2.2	3.3	2.8	3.8	4.3
50–<100%	8.3	5.8	7.1	8.1	9.2	11.5
100%	88.4	91.9	89.6	89.1	87.0	84.2
On-T SBP, mmHg	135.2 ± 14.3	135.6 ± 13.4	135.2 ± 14.0	135.2 ± 14.2	134.8 ± 14.7	135.4 ± 15.0
On-T DBP, mmHg	77.7 ± 8.1	78.7 ± 7.6	78.1 ± 7.9	77.5 ± 8.1	77.2 ± 8.2	76. 8 ± 8.5
On-T HR, bpm	68.9 ± 9.4	69.5 ± 8.8	69.1 ± 9.4	68.6 ± 9.4	68.6 ± 9.5	68.5 ± 9.8

Data are shown as mean ± standard deviation or %. BP and HR values during treatment are included. BMI, body mass index; CV, coefficient of variation; DBP, diastolic blood pressure; eGFR, estimated glomerular filtration rate (MDRD formula); HR, heart rate; MI, myocardial infarction; SBP, systolic blood pressure; T, treatment; TIA, transient ischemic attack.

In addition to the conventional calculation of SBP-CV (as described above), we calculated an alternative measure of visit-to-visit SBP variability which takes into account the seasonal changes over the year. Instead of the deviations between the measurements and the individual mean, we took the deviations to the individual mean adjusted for the expected seasonal effect as the basis for calculating the standard deviation. The difference between the two approaches is described in the following example: let's assume the measured SBP value taken in August is 130 mmHg, and the individual mean SBP (over five visits) is 135 mmHg. In the conventional way of calculating the SBP-CV the contribution of this individual measurement is 130 − 135 = −5. However, from the analysis across all patients the average SBP in August shows a reduction of 2.4 mmHg. Therefore we based the calculation of the alternative SBP-CV on the difference between the measured and expected value (when seasonality is taken into account), i.e. 130 − (135 – 2.4) =  −2.6. In the above example the contribution of the individual SBP measurement during summer leads to a reduction of SBP-CV, which is in line with the usually lower SBP values during summer. However, SBP-CV can also be increased by a SBP summer individual measurement if its value is in contrast to the seasonal pattern. To emphasize that the common effect of seasonality is subtracted from the conventional or original SBP-CV we called SBP-CV after removal of seasonality residual SBP-CV.

Finally, in order to assess the impact of seasonal SBP changes on the prognostic relevance of visit-to-visit BP variability we calculated the association of SBP-CV quintiles with the primary and the secondary outcomes occurring during the roughly 3.5 years after the initial 2-year period necessary to quantify visit-to-visit SBP-CV over five visits. Outcomes were related to both the conventional SBP-CV and to the residual SBP-CV via the Cox regression model, using quintiles of SBP-CV and the SBP-CV values directly as a linear variable; data were always adjusted for the covariates displayed in Table 1. Adjustment included the four treatment arms from the two trials and was extended to the adherence to treatment (which had been measured by pill counting) and to the on-treatment 2-year mean SBP values. Despite our previous demonstration that in the ONTARGET-TRANSCEND population there is no association between SBP-CV and mean SBP (12, see above) we thought that the latter adjustment further guaranteed the exclusive dependence of the results on SBP variability with no concomitant contribution of mean SBP values. Time-to-event data were shown in Kaplan–Meier curves, and results of the Cox models were expressed as hazard ratios with 95% confidence intervals for conventional and residual SBP-CV. Between-quintile differences were shown using the 1st quintile as reference, while for the linear models hazard ratios were shown for an increase of 10 units. The validity of the proportional hazard assumption was checked using the Schoenfeld residuals. Comparison between models was done by the Vuong test. Further methodological details are available in previous publications [12,13,44]. Throughout the text the symbol ± refers to the SD or the standard error of the mean. A *P* < 0.05 was taken as the level of statistical significance.

## RESULTS

### Demographic and clinical data

Data were collected from the centers reported in Table S1, Supplemental digital Content. The demographic and clinical characteristics of the patients analyzed for the monthly BP values and for the association between BP variability and cardiovascular and mortality outcomes are shown in Table 1. Briefly, many more patients were recruited from the northern than from the southern hemisphere. Average age was around 66 years and males represented about 70% of the study population. At baseline mean SBP was >140 mmHg whereas mean DBP was <90 mmHg. Hypertension was present in about 70% of the patients, diabetes in about one third, obesity in about one third, and previous cardiovascular events were reported in a variable proportion of patients, i.e. from about 50% (previous myocardial infarction) to 20% or less (stroke). Both SBP and DBP were lower during the BP variability quantification period than at baseline. Patients were more frequently treated with telmisartan than with ramipril and a limited number of patients (all from the TRANSCEND trial) was on placebo. Most variables were similar between SBP-CV quintiles which exhibited nearly superimposable mean BP values.

### Seasonal BP changes

**FIGURE 1:**
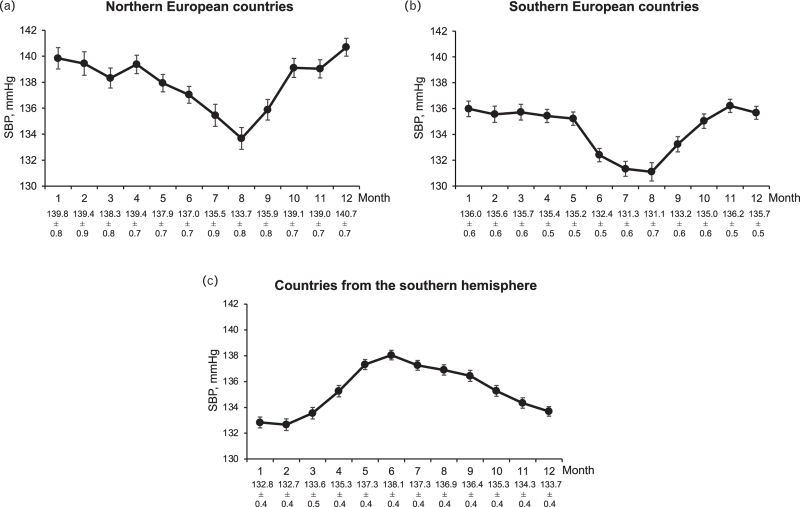
Monthly (January to December) systolic blood pressure (SBP) in 25 390 patients of the ONTARGET and TRANSCEND trials, separately for patients from northern European countries, southern European countries and patients from the southern hemisphere who as expected showed an inverse seasonality. Data at the bottom refer to monthly SBP means ± standard errors.

**FIGURE 2:**
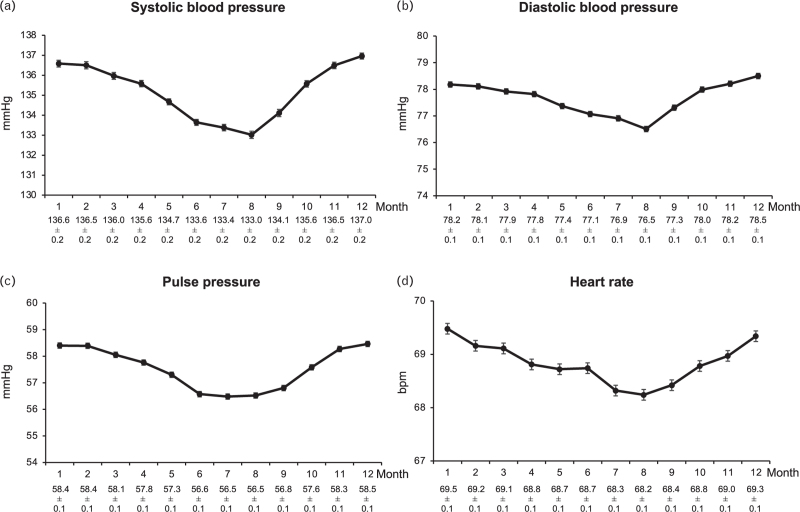
Monthly systolic BP, diastolic BP, pulse pressure and heart rate values in the patients of Fig. 1 pooled (*n* = 25 390). Data of patients from the southern hemisphere were corrected for inverse seasonality (see Methods). Explanations and abbreviations as in Fig. 1.

**FIGURE 3:**
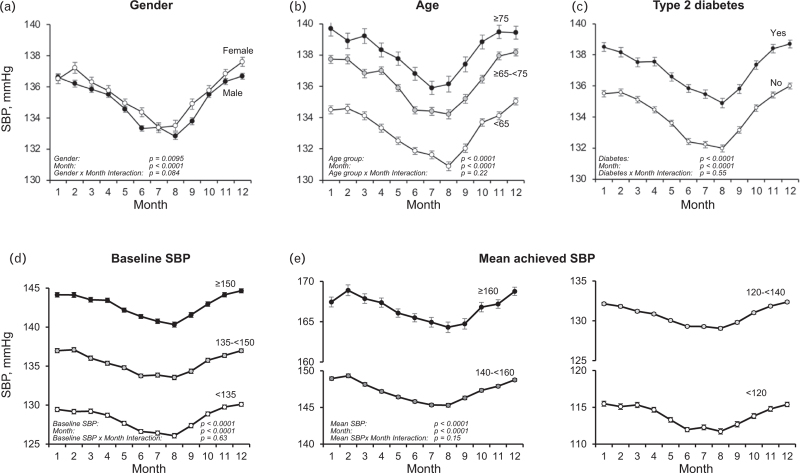
Monthly systolic blood pressure (SBP) values according to patients’ gender, age, presence or absence of type 2 diabetes mellitus, baseline SBP and mean achieved SBP during the treatment period. Data refer to patients of Figs. 1 and 2. Data from the southern hemisphere were corrected for inverse seasonality. Baseline and achieved SBP ranges are indicated in the panels. Data from different achieved SBP ranges are shown in the central and right bottom panels. Abbreviations and explanations as in preceding figures.

Figure 1 shows the mean SBP values, separately in patients from countries in northern Europe (Denmark, Norway, Finland, Sweden, *n* = 1417), southern Europe (Italy, Greece, Spain, Turkey, *n* = 1770), and the southern hemisphere (Argentina, Brazil, South Africa, Australia, New Zealand, *n* = 4752). Mean SBP values in northern Europe were consistently about 4 mmHg higher compared to southern Europe. Patients from the northern hemisphere showed a progressive SBP reduction from January to July and a subsequent increase from July to December while the opposite was the case in patients from the southern hemisphere. When data were corrected for the inverse seasonality and adjusted for the covariates shown in Table 1, the SBP reduction from the month with the highest BP in winter and the month with the lowest BP in summer amounted to about 4 mmHg, the corresponding diastolic BP, pulse pressure and HR reductions amounting to about 2 mmHg, 2 mmHg and 2 beats/min (Fig. 2). Although the significance of several *P* values for subgroup-by-month interaction indicated that the effects of seasonality were not identical between subgroups, the adjusted winter-summer SBP pattern was similar in males and females, younger and older patients, diabetic and nondiabetic patients, patients with different baseline SBP values and patients with different achieved on-treatment mean SBP values (Fig. 3).

### Seasonal BP changes and visit-to-visit SBP variability

**FIGURE 4:**
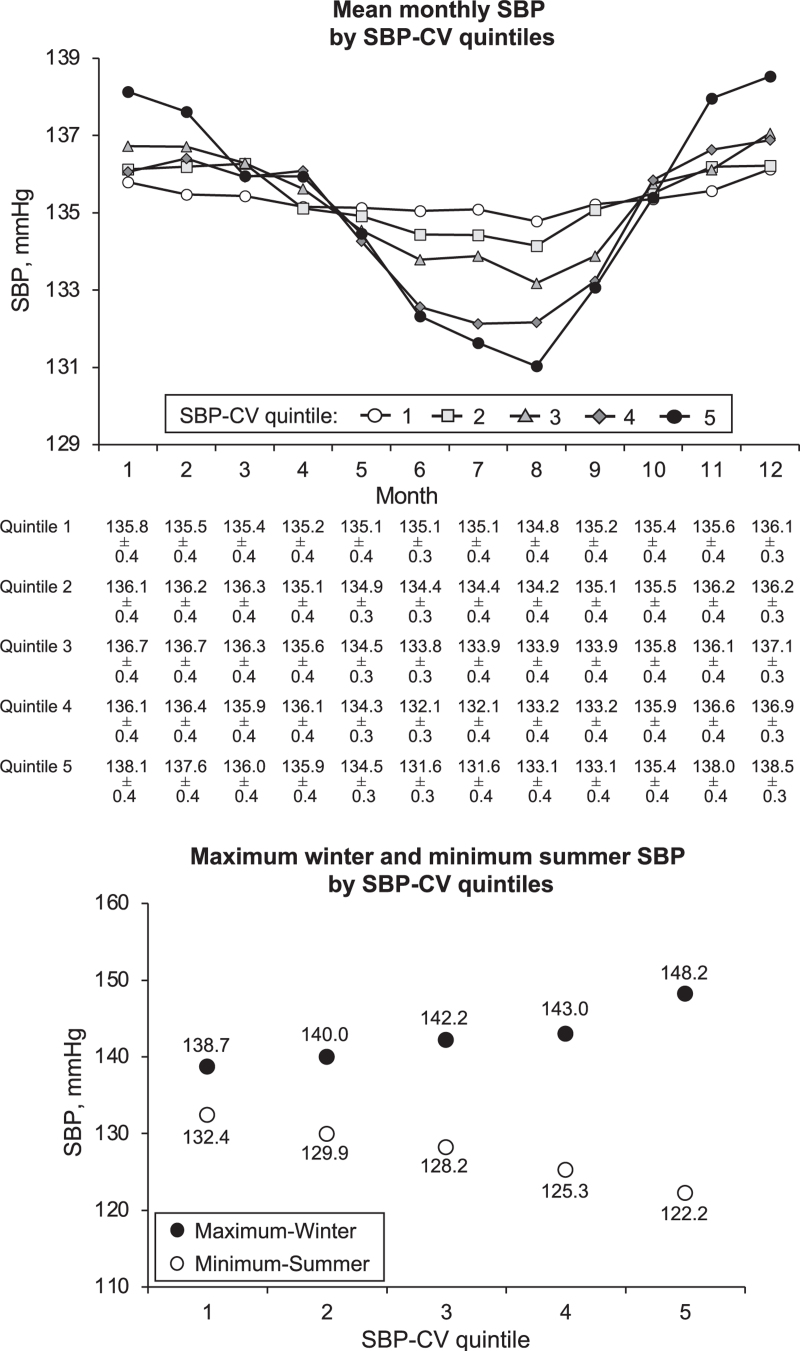
The upper panel shows the mean monthly SBP values in different SBP-CV quintiles, i.e. from the lowest (1) to the highest one (5). The lower panel shows the mean maximum winter and mean minimal summer SBP values according to the SBP-CV quintile 1 to 5. Data from the 25 390 patients of the preceding Figures. Abbreviations as in preceding figures.

Figure 4, upper panel, shows that in all SBP-CV quintiles mean SBP (corrected for inverse seasonality and adjusted for the variables listed in Table 1 as well as for on-treatment mean SBP, see Methods) was higher in winter than in summer months. From the quintile with the smallest to the quintile with the greatest SBP-CV (quintiles 1 to 5) SBP exhibited progressively higher values in winter months and progressively lower values in summer months, indicating that an increase of visit-to-visit SBP variability was associated with a progressively greater seasonal effect on BP. As shown in the lower panel of Fig. 4, this was accompanied by a progressive expansion of the seasonal-related BP range, i.e. from the 1st to the 5th SBP-CV quintile the mean maximum SBP value registered in the winter months exhibited a progressive increase while the mean minimal SBP value registered in the summer months exhibited a progressive reduction, indicating a progressively greater seasonal-related BP dispersion as visit-to-visit SBP variability increased. As shown in Table S2, Supplemental Digital Content the difference between conventional and residual SBP-CV became progressively greater from quintile 1 to quintile 5. Thus, the contribution of seasonality increased progressively with the increase of SBP-CV, the change being virtually always significant between quintiles.

### Seasonal BP changes and prognostic value of visit-to-visit SBP variability

**FIGURE 5:**
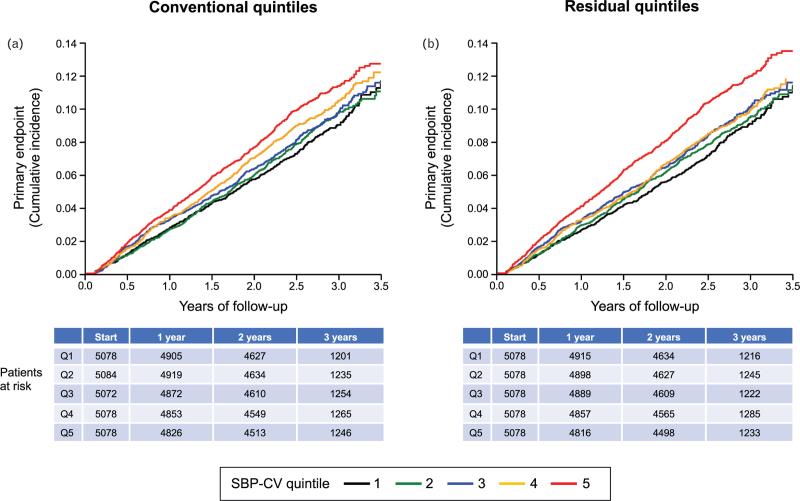
Progressive increase in incidence of the primary end-point (Kaplan–Meier curves, cardiovascular morbidity and mortality) for each SBP-CV quintile before (conventional) and after (residual) subtraction of seasonal SBP changes. Event incidence was progressively greater from the lowest to the highest SBP-CV quintile. Quintile differences became apparent early after treatment initiation. For each quintile data were corrected for inverse seasonality and adjusted for the variables of Table 1 (see Methods). Quintiles are numbered from 1 to 5.

**FIGURE 6:**
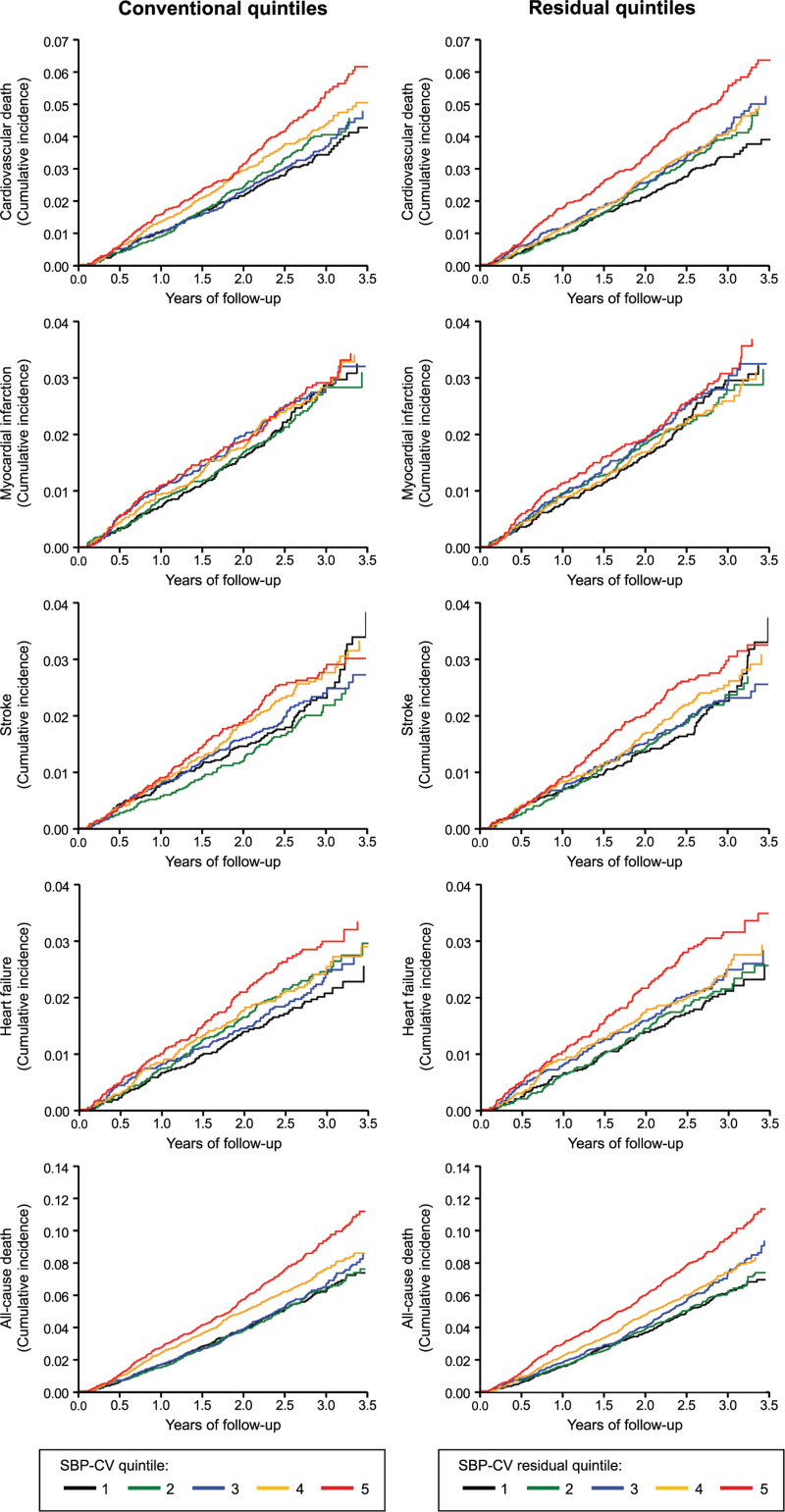
Progressive increase in the incidence of secondary end-points (Kaplan–Meier curves) for each SBP-CV quintile before (conventional) and after (residual) subtraction of seasonal BP changes in patients of Fig. 5. MI, myocardial infarction; HF, heart failure. Other explanations as in Fig. 5.

As shown in Fig. 5 and Table 2, either without (conventional) and after (residual) subtraction of seasonal SBP changes the incidence of the primary endpoint (Kaplan–Meier curves, left and right panels, respectively) increased progressively from SBP-CV quintile 1 to quintiles 2, 3, 4 and 5, the number of events (and yearly event rates) being 427 (3.17%), 450 (3.32%), 462 (3.42%), 499 (3.71%) and 543 (4.09%) in conventional quintiles, and, respectively, 427 (3.16%), 444 (3.28%), 467 (3.47%), 480 (3.56%) and 563 (4.24%) in residual quintiles. The separation between quintiles became visible in a relatively early phase of the observation period and the cumulative incidence showed an especially marked increase in the two highest SBP-CV quintiles. Similar results for both the cumulative incidence and the yearly event rate were obtained for the Kaplan-Meier curves related to secondary endpoints (Fig. 6 and Table 2a).

**TABLE 2:** Hazard ratios and 95% confidence intervals (CI) for the primary and secondary endpoints of the ONTARGET and TRANSCEND trials according to quintiles of SBP-CV (conventional and residual). Quintile 1 is taken as reference (Ref). *P*-values (trend) refer to the hazard ratios from quintile 1 to 5. Comparisons between models was made by the Vuong test. Hazard ratios are adjusted for confounders shown in TABLE 1 (using values at the end of the 2-year quantification period whenever appropriate)

	Quintiles yearly event rate/hazard ratio (95% CI)						
Parameter	1	2	3	4	5	*P*-value (trend)	Vuong-test (conv. vs. resid.)
	Primary endpoint						
SBP-CV conventional	*3.17* Ref	*3.32* 1.06 (0.93–1.21)	*3.42* 1.06 (0.93–1.21)	*3.71* 1.12 (0.98–1.27)	*4.09* 1.16 (1.02–1.32)	0.21	0.32
SBP-CV residual	*3.16* Ref	*3.28* 1.03 (0.90–1.18)	*3.47* 1.07 (0.94–1.22)	*3.56* 1.08 (0.94–1.23)	*4.24* 1.20 (1.05–1.36)	0.060	
	Cardiovascular death						
SBP-CV conventional	*1.21* Ref	*1.36* 1.14 (0.93–1.41)	*1.27* 1.04 (0.84–1.29)	*1.52* 1.18 (0.97–1.45)	*1.79* 1.31 (1.07–1.60)	0.062	0.78
SBP-CV residual	*1.18* Ref	*1.31* 1.12 (0.91–1.38)	*1.39* 1.16 (0.94–1.43)	*1.44* 1.16 (0.94–1.43)	*1.83* 1.36 (1.12–1.67)	0.041	
	Myocardial infarction						
SBP-CV conventional	*0.92* Ref	*0.89* 0.97 (0.76–1.25)	*0.98* 1.07 (0.84–1.36)	*0.98* 1.05 (0.82–1.34)	*1.01* 1.05 (0.82–1.34)	0.95	0.97
SBP-CV residual	*0.95* Ref	*0.88* 0.93 (0.72–1.19)	*0.94* 0.98 (0.77–1.26)	*0.98* 1.01 (0.79–1.29)	*1.03* 1.03 (0.81–1.32)	0.93	
	Stroke						
SBP-CV conventional	*0.82* Ref	*0.70* 0.87 (0.66–1.13)	*0.81* 0.96 (0.74–1.24)	*0.94* 1.10 (0.86–1.42)	*0.98* 1.08 (0.84–1.39)	0.48	0.50
SBP-CV residual	*0.79* Ref	*0.77* 0.97 (0.74–1.27)	*0.81* 0.99 (0.76–1.30)	*0.87* 1.06 (0.81–1.37)	*1.02* 1.16 (0.90–1.48)	0.66	
	HF hospitalization						
SBP-CV conventional	*0.69* Ref	*0.85* 1.24 (0.94–1.62)	*0.78* 1.10 (0.84–1.46)	*0.85* 1.11 (0.84–1.46)	*1.03* 1.24 (0.95–1.61)	0.48	0.83
SBP-CV residual	*0.69* Ref	*0.79* 1.13 (0.86–1.49)	*0.80* 1.13 (0.86–1.48)	*0.82* 1.09 (0.83–1.43)	*1.09* 1.29 (1.00–1.68)	0.39	
	All-cause mortality						
SBP-CV conventional	*2.17* Ref	*2.17* 1.00 (0.85–1.18)	*2.27* 1.01 (0.86–1.18)	*2.61* 1.11 (0.95–1.29)	*3.24* 1.28 (1.10–1.49)	0.0018	0.40
SBP-CV residual	*2.14* Ref	*2.10* 0.98 (0.83–1.15)	*2.40* 1.08 (0.93–1.27)	*2.53* 1.10 (0.94–1.18)	*3.28* 1.31 (1.13–1.52)	0.0005	

Abbreviations as in the preceding table.

As shown in Table 2a, first two rows, the adjusted hazard ratios for the primary endpoint resulting from the Cox regression analysis showed a progressive increase of risk from quintiles 1 to 5 for both quintile-based (conventional and residual) calculation of SBP-CV. For each quintile, the hazard ratio was usually modestly greater after than before subtraction of seasonal BP changes. The global trend test did not confirm a statistically significant difference between the conventional quintiles (*P* = 0.21), while for the residual quintiles the differences were marginally significant (*P* = 0.060). As shown in Table 3, the alternative Cox model, in which SBP-CV was analyzed as a linear variable (instead of classifying subjects into quintiles), showed that the hazard increases significantly with increasing SBP-CV, both for the conventional (*P* = 0.0070) and for the residual (*P* = 0.0021) measures of visit-to-visit SBP variability. In addition, the Vuong-test (which tests which of the two models, conventional vs residual, is closer to the true model) indicated that the residual model is to be preferred. Of note, the results were not noticeably affected by adjustment for adherence or trial arm. The Schoenfeld residuals did not show any relevant deviation from a zero-slope if plotted against event time, thus confirming that the proportional hazards assumption, which is a prerequisite of the Cox model, was justified.

**TABLE 3:** Hazard ratios and 95% confidence intervals (CIs) for the primary and secondary endpoints of the ONTARGET and TRANSCEND trials according to SBP-CV (conventional and residual) in linear models. Comparisons between conventional and residual models was made by the Vuong test. Hazard ratios are adjusted for confounders shown in Table 1 and are given for an increase of 10 units

Parameter	Hazard Ratio (95% CI) for an increase of 10 units	P-value	Vuong-test (conv. vs resid.)	Vuong-test (vs quintile model)
	Primary endpoint			
SBP-CV conventional	1.14 (1.04–1.26)	0.0070	0.048	0.56
SBP-CV residual	1.17 (1.06–1.29)	0.0021		0.81
	Cardiovascular death			
SBP-CV conventional	1.23 (1.06–1.42)	0.0070	0.078	0.63
SBP-CV residual	1.25 (1.08–1.45)	0.0027		0.73
	Myocardial infarction			
SBP-CV conventional	1.09 (0.90–1.31)	0.39	0.52	0.98
SBP-CV residual	1.10 (0.91–1.33)	0.33		0.95
	Stroke			
SBP-CV conventional	1.16 (0.95–1.41)	0.14	0.99	0.56
SBP-CV residual	1.16 (0.95–1.42)	0.14		0.95
	HF hospitalization			
SBP-CV conventional	1.13 (0.93–1.37)	0.23	0.21	0.55
SBP-CV residual	1.17 (0.97–1.43)	0.11		0.56
	All-cause mortality			
SBP-CV conventional	1.27 (1.13–1.42)	<0.0001	0.021	0.83
SBP-CV residual	1.29 (1.16–1.45)	<0.0001		0.83

Abbreviations as in the preceding figure.

Among the secondary endpoints, the results were similar to the primary endpoint for all-cause mortality, and to a lesser degree for cardiovascular mortality, but even clearer and throughout significant. With a quintile-based increase of SBP-CV, both conventional and residual, the risk of all-cause death increased significantly (*P* = 0.0018 for conventional and *P* = 0.0005 for residual). This was the case also for the linear model (*P* < 0.0001 for both). The Vuong-test comparing the two linear models was also significant (*P* = 0.021) suggesting that the residual SBP-CV delivers a more accurate measure of the risk associated with an increase of visit-to-visit BPV. For the other secondary endpoints (MI, stroke, HF hospitalization) no association with BP variability was seen, and this applied to both the conventional and the residual SBP-CV.

## DISCUSSION

Our study on a large number of patients treated with antihypertensive drugs for 5 years or more shows that visit-to visit SBP variability originates in part from seasonal SBP differences, i.e. from the difference between the lower BP values that occur during summer and the higher ones that occur during winter time. It also shows that the contribution of BP seasonality to visit-to-visit SBP variability increases progressively as the size of visit-to-visit SBP variability increases. Thus, in patients under antihypertensive treatment visit-to-visit SBP variations are induced not only by pathophysiological or clinically-dependent factors such as between-visit differences in adherence to the prescribed treatment regimen [16,17], use of specific antihypertensive drugs [18–24], severity of organ damage or alterations of mechanisms involved in cardiovascular modulation [25–30], but also by physiological factors such as seasonal-related SBP modifications.

The evidence that visit-to-visit SBP variability originates not only from pathophysiological and clinical factors but also from physiological factors such as seasonal SBP variations leads to a further important question that was central to our study. That is, whether this physiological component affects the adverse prognostic significance of visit-to-visit SBP variability [4–15]. In an attempt to shed light on this question we assessed the relationship between visit-to-visit SBP variability and the risk of cardiovascular outcomes or mortality before and after subtracting the seasonal SBP component from the original or conventional visit-to visit SBP variability value. In either condition visit-to-visit SBP variability exhibited an adverse prognostic significance, i.e. its increase was associated with an increase of cardiovascular outcomes and mortality both before and after subtraction of the seasonal SBP component. However, after subtraction of the seasonal component the relationship between increase in SBP variability and the risk of outcomes such as overall cardiovascular morbidity and mortality (the primary endpoint of the trials) or all-cause mortality became steeper, the difference from the presubtraction relationship reaching statistical significance with use of the linear model of SBP variability. This allows to conclude that the seasonal component is not responsible for the adverse prognostic significance of visit-to-visit SBP variability, which thus is more likely to be accounted for by the pathophysiological and clinical factors that have been reported as a cause of this phenomenon. It further allows to suggest, however, that, as far as the risk associated with visit-to-visit BP variations is concerned, seasonal SBP changes are not entirely neutral but may rather play an attenuating role, which is revealed by the steeper relationship of visit-to-visit SBP variability with cardiovascular outcomes and mortality when the seasonal component of SBP variability is removed.

The BP differences between summer and winter time have been reported by many studies [37], and are known to extend to BP values obtained outside the physician's office, i.e. also when BP is self-measured at home or over the 24 h [32,36,37,39,45]. Our investigation adds to previous knowledge evidence of SBP seasonality from a large number of patients under antihypertensive treatment living in widely different parts of the world and exposed to widely different climates, which documents. that this phenomenon involves different demographic and clinical conditions, thus having an universal distribution. The factors involved in seasonal BP differences were not addressed by our study because the trials from which data were retrieved did not collect relevant information. However, they have been the object of other investigations which have shown a role of lower indoor or outdoor temperature in the higher winter BP levels [33,37,46–51] as well as of physical activity, greater socialization and reduction of work-dependent stress in the lower summer BP values [38]. These factors may operate at least in part via modulation of sympathetic activity which has been shown to increase as environmental temperature is reduced [52], and to be greater in sedentary people and in response to stress [51,53,54]. In this context, it is relevant to mention that in our study summer was accompanied by a small but significant reduction of heart rate. Because of the concomitant BP reduction this did not have a baroreflex origin and thus probably reflected a central alteration of cardiac autonomic control, such as a reduction of cardiac sympathetic and/or an increase of vagal drive.

Our study has several elements of strength but also some weaknesses. The elements of strength are the originality of the research question addressed, the high number of patients studied and events collected, the identical number of visits available for the visit-to-visit SBP variability quantification and the appropriateness of the study design which quantified SBP variability first and risk of outcome later. It should also be mentioned that the prognostic value of visit-to-visit SBP variability was assessed by a measure (SBP-CV) independent from on-treatment mean SBP, that data were further adjusted for mean SBP values and that, most importantly, variability quintiles had superimposable mean SBP values (see Table 1). Thus, the present study ensures that the BPV-outcome relationship was investigated without the confounding effect of mean BP on the prognostic value of BP variability as it is the case in several other studies using standard deviation or derived indices to quantify variability [12]. Weaknesses are that BP was measured only once at each visit, possibly with an amplifying effect on visit-to-visit BP variations. Furthermore, our data on SBP seasonality have only a descriptive value, with no insight into the factors involved in this phenomenon. Because the seasonal SBP differences can have disparate reasons in different individuals their prognostic role may vary according to the factors more or less importantly involved. This may lead to individual variations in the influence of seasonality on the prognostic role of visit-to-visit SBP variability, a phenomenon that cannot be taken into account by population mean values. Finally, our evidence refers primarily to patients treated with blockers of the renin-angiotensin system and whether the same conclusion applies to patients treated with other drugs remains to be assessed.

## ACKNOWLEDGEMENTS

None.

Funding: ONTARGET and TRANSCEND trials were funded by a grant from Boehringer Ingelheim. ONTARGET and TRANSCEND studies are registered with ClinicalTrials.gov NCT00153101 and NCT0263104, respectively. Boehringer Ingelheim did not fund or was involved in the present study

### Conflicts of interest

The corresponding author has nothing to disclose with regard to the present paper. This is the case also for the remaining authors.

Data availability statement: The data underlying this article will be shared on reasonable request to the corresponding author.

## Supplementary Material

Supplemental Digital Content
