# Supplementary material for: Impact of seasonal blood pressure changes on visit-to-visit blood pressure variability and related cardiovascular outcomes
Source: J Hypertens. 2024 May 1;42(7):1269–81. doi: 10.1097/HJH.0000000000003759 (PMC11198955; doi:10.1097/HJH.0000000000003759)
Supplement: Supplemental Digital Content [file jhype-42-1269-s001.pdf]

SUPPLEMENTARY MATERIAL

IMPACT OF SEASONAL BLOOD PRESSURE CHANGES
ON VISIT-TO-VISIT BLOOD PRESSURE VARIABILITY AND RELATED
OUTCOMES

Giuseppe Mancia, MD1; Helmut Schumacher, PhD2; Michael Böhm, MD3;
Guido Grassi, MD1; Teo K Koon, PhD4; Felix Mahfoud, MD3;
Gianfranco Parati, MD1; Josep Redon, MD5 and Salim Yusuf, DPhil4.

Supplementary Table 1. Number of patients in participating countries

**Total**		**25390**		
Northern Hemisphere		20638		
	Europe		11590	
	Austria			125
	Belgium			752
	Czechia			548
	Denmark			406
	Finland			325
	France			701
	Germany			1445
	Greece			191
	Hungary			544
	Ireland			149
	Italy			903
	Netherlands			957
	Norway			213
	Poland			710
	Portugal			171
	Russia			518
	Slovakia			356
	Spain			485
	Sweden			473
	Switzerland			192
	UK			791
	Ukraine			635
	North America		4719	
	Canada			2360
	USA			2359
	Latin-America		382	
	Mexico			382
	Asia / Middle East		3947	
	China			1460
	Hong Kong			355
	Malaysia			237
	Philippines			279
	Singapore			68
	South Korea			364
	Taiwan			425
	Thailand			366
	Turkey			191
	UAE			202
Southern Hemisphere		4752		
	Australia / New Zealand		1747	
	Australia			1105
	New Zealand			642
	South-America		2351	
	Argentina			1389
	Brazil			962
	Africa		654	
	South Africa			654

Supplementary Table 2. Mean contribution to each SBP-CV quintile by SBP seasonality and P values of between-quintile different contributions.

Quintile	Mean change (95% CI) Conventional – Residual SBP-CV	p-value vs 1st quintile	p-value vs 2nd quintile	p-value vs 3rd quintile	p-value vs 4th quintile
1	0.055 (0.041, 0.069)				
2	-0.036 (-0.050, -0.021)	< 0.0001			
3	-0.085 (-0.099, -0.077)	< 0.0001	< 0.0001		
4	-0.099 (-0.114, -0.086)	< 0.0001	< 0.0001	0.67	
5	-0.124 (-0.138, -0.110)	< 0.0001	< 0.0001	0.0012	0.099

Data from patients of Table 1. Abbreviations as in preceding Figures and Table 1.
